# GLP-1 Receptor Agonists Associated With Improved Survival After Infrainguinal Bypass in Diabetic Patients

**DOI:** 10.1016/j.avsg.2025.08.013

**Published:** 2025-08-16

**Authors:** Elonay Yehualashet, Muhammad S. Mazroua, Estefania Narvaez, Marissa C. Jarosinski, Nathan L. Liang, Michael C. Madigan, Rabih A. Chaer, Natalie D. Sridharan

**Affiliations:** Division of Vascular Surgery, University of Pittsburgh Medical Center, Pittsburgh, PA.

## Abstract

**Background:**

The American Diabetes Association recommends the use of glucagon-like-peptide-1 receptor agonists (GLP1-RAs) (e.g., Ozempic) for patients with atherosclerotic cardiovascular disease, including peripheral artery disease. We hypothesized increasing the use of GLP1-RAs would be associated with improved outcomes after bypass.

**Methods:**

Retrospective review of diabetic patients undergoing infrainguinal bypass at a multi-hospital institution between 2017 and 2024 was conducted, including patient characteristics, operative details, and outcomes. The primary outcome was survival. Secondary outcomes included perioperative complications, major adverse limb events, major adverse cardiovascular events (MACE), and metabolic changes. Kaplan–Meier and Cox regression evaluated outcomes.

**Results:**

A total of 322 patients (62.5% male, 78.8% White, median age 68 [61–76] years) underwent 339 bypasses. GLP1-RA use increased from 8.2% to 18.8% during the study period (*P* = 0.001). Patients on a GLP1-RA (*n* = 38, 11.2%) were younger (65.5 [57.0–71.0] vs. 69.9 [62.0–77.0] years, *P* = 0.003) and had a higher body mass index (29.6 [26.9–33.3] vs. 27.2 [23.3–31.1] kg/m^2^, *P* < 0.001). Median follow-up was 18 [6–39] months. Improved survival at 2 years was seen among patients on a GLP1-RA (94.7% vs. 71.8%, *P* = 0.004). On adjusted analysis, GLP1-RA use was associated with reduced mortality (adjusted hazard ratio = 0.09, 95% confidence interval 0.01–0.66, *P* = 0.02). Freedom from MACE at 2 years was greater among patients on a GLP-1-RA (100% vs. 88.6%, *P* = 0.046). Freedom from major adverse limb events was similar between groups.

**Conclusion:**

GLP1-RA use is increasingly common in patients undergoing lower extremity bypass and was associated with significantly improved postoperative survival and freedom from MACE. Further studies are required to confirm reasons for differences in outcomes.

## INTRODUCTION

In the United States, approximately 50% of patients with type 2 diabetes (T2DM) have atherosclerotic cardiovascular disease (ASCVD).^1^ Peripheral artery disease (PAD), a common manifestation of ASCVD, affects over 220 million people globally and is increasingly prevalent due to an aging population, diabetes, and obesity.^2,3^ Glucagon-like peptide-1 receptor agonists (GLP-1 RAs) were first approved in 2005 for glycemic control in patients with T2DM. More recently, GLP-1 RAs have been found to provide cardiovascular benefits, similar to sodium-glucose cotransporter 2 inhibitors. Several cardiovascular outcome trials (CVOTs) have established the efficacy of GLP-1 RAs in reducing cardiovascular disease risk in T2DM patients, demonstrating a reduction in major adverse cardiovascular events (MACE) by up to 26% with GLP-1 RA use.^4–10^ Based on these findings, the American College of Cardiology published a recent decision pathway for the use of novel therapies for cardiovascular risk reduction in patients with T2DM. In this pathway, both the European and United States professional societies recommended that GLP-1 RAs be considered as a first-line therapy for all patients with T2DM and either an elevated cardiovascular disease risk or established ASCVD, including PAD.^11–13^ Despite this, PAD has not been specifically studied in the CVOTs. In addition, limb outcomes have not been assessed in these trials. Therefore, we compared outcomes in diabetic patients undergoing infrainguinal bypass for PAD, hypothesizing that the use of a GLP-1 RA would be associated with improved postoperative survival, likely through a reduction in adverse cardiovascular events.

## METHODS

### Data Source and Study Cohort

The cohort included all adult diabetic patients who underwent infrainguinal bypass for PAD at a multi-hospital health care system between January 2019 and December 2024. Patients with a prior ipsilateral bypass or a history of end-stage renal disease were excluded, as GLP-1 RAs are contraindicated in end-stage renal disease. Patient demographics, medications, operative details, and outcomes were obtained from the electronic medical record. The final cohort was stratified into 2 groups based on the presence of a GLP-1 RA prescription at the time of surgery. All Food Drug Administation–approved, commercially available GLP-1 RAs were considered, including semaglutide, tirzepatide, dulaglutide, and liraglutide. The University of Pittsburgh’s human research protection office approved the present study as a nonhuman research (IRB PRO #24070120).

### Variable Definitions and Outcomes

The primary outcome was overall survival. Secondary outcomes included freedom from MACE, defined as a composite of cardiovascular death, nonfatal myocardial infarction, or nonfatal stroke; freedom from major adverse limb events (MALE), defined as a composite of major reintervention or above-ankle amputation on the ipsilateral limb; and changes in metabolic parameters, including body mass index (BMI), hemoglobin A1c, glucose, and creatinine. A major reintervention was defined as either a revision of the bypass or thrombectomy (open or percutaneous) of the index arterial segment. For the purpose of this study, optimal medical therapy (OMT) was defined as the preoperative use of statins and antiplatelet medications and nonsmoking status. The primary outcome of survival was evaluated in the entire cohort, including in-hospital deaths, whereas the secondary outcomes were evaluated only in patients who survived index hospitalization and were available for follow-up.

### Statistical Analysis

Baseline characteristics were obtained for the overall cohort and compared between groups. Continuous variables are presented as means with standard deviation or medians with interquartile range and are compared using the *t*-test or Mann–Whitney *U*-test, based on the distribution of the data. Categorical variables are presented as frequencies with percentages and are compared using the chi-square (χ2) or Fisher exact tests. The outcomes were evaluated using Kaplan–Meier estimates and the log-rank test. A multivariate Cox regression model was used to identify independent predictors of the outcomes and quantify treatment effects using adjusted hazard ratios (aHRs) with 95% confidence intervals (CIs). A threshold value of *P* < 0.05 on the univariate analysis was used to screen covariates for inclusion in the regression model. Additional covariates were included based on clinical relevance and significant differences in baseline characteristics, such as age, BMI, and indication (claudication vs. chronic limb-threatening ischemia [CLTI]). A sensitivity analysis was performed, modifying the definition of MALE to include any vascular reintervention of the index arterial segment. Lastly, a subgroup analysis was performed on patients with at least 2 components of OMT to evaluate whether OMT had a modulating effect on the primary outcome of survival. Data analysis and figure generation were completed using Stata 17.0 (StataCorp LP, College Station, TX).

## RESULTS

### GLP-1 RA Trends and Patient Characteristics

**Fig. 1.**
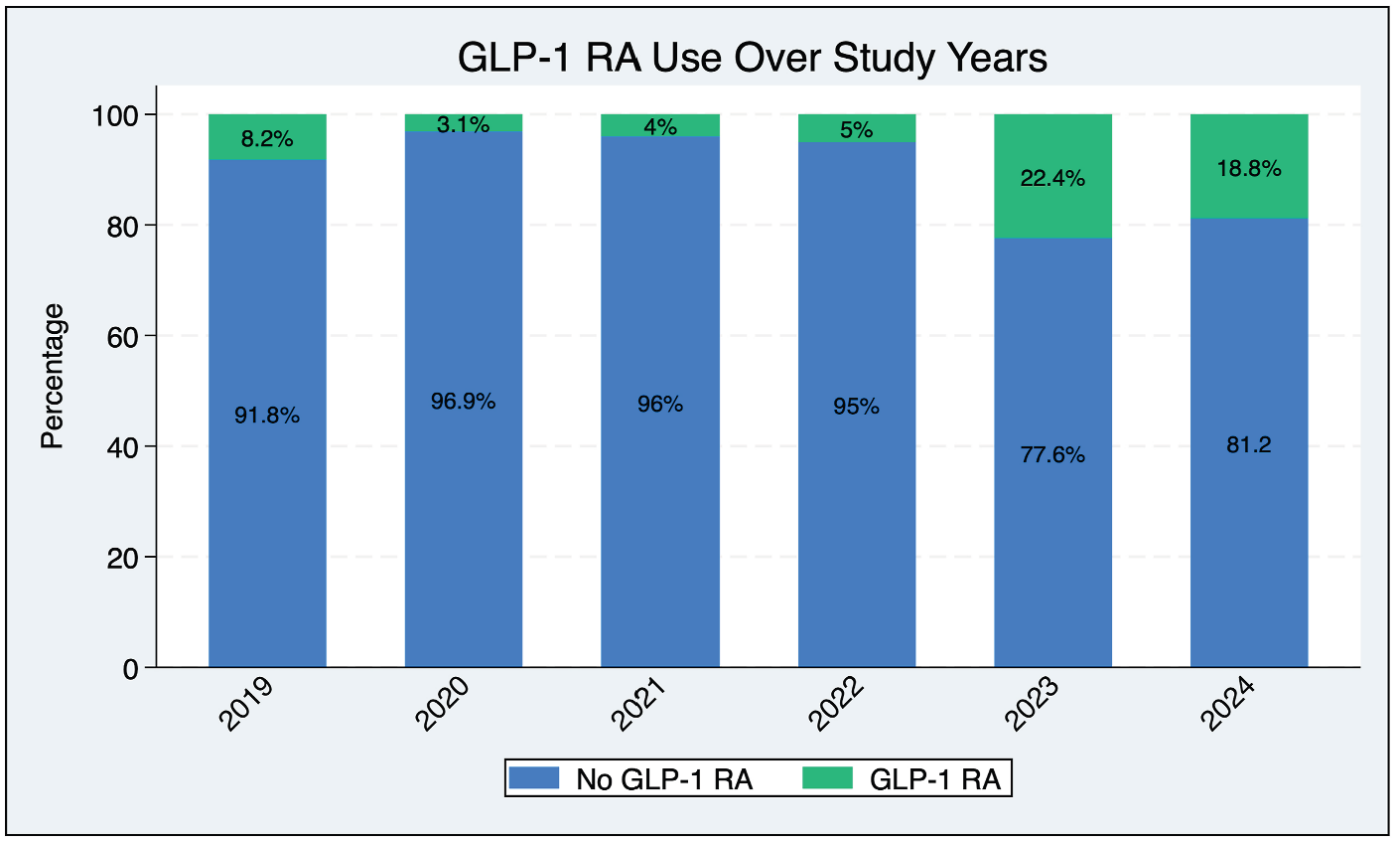
GLP-1 RA use over study years. The proportion of diabetic PAD patients undergoing infrainguinal bypass who were prescribed a GLP-1 RA increased significantly from 8.2% in 2017 to 18.8% in 2024 (*P* = 0.001).

A total of 322 patients undergoing 339 bypass procedures were included in the analysis. Of these, approximately 11% (*n* = 38) were on a GLP-1 RA at the time of surgery (Table I). Over the study period, there was a trend toward increased use of GLP-1 RAs, with the proportion of patients on a GLP-1 RA more than doubling over the years (8.2%–18.8%, *P* = 0.001) (Fig. 1). The overall cohort was predominantly male (62.5%) and White (78.8%) and presented with CLTI (94.6%). Comorbid conditions were common, including hypertension (95.2%), coronary artery disease (51.3%), and congestive heart failure (22.7%). Patients on a GLP-1 RA were younger (65.5 [57.0–71.0] vs. 69.9 [62.0–77.0] years, *P* = 0.003) and had a higher BMI (29.6 [26.9–33.3] vs. 27.2 [23.3–31.1] kg/m^2^, *P* < 0.001). Preoperative use of statins, antithrombotic medications, and nonsmoking status was similar between the groups. Additional baseline characteristics are included in Table I.

### Operative Details and In-Hospital Outcomes

Operative details and in-hospital outcomes are summarized in Table II. There were no significant differences between the groups in indication for intervention, level of disease treated, procedure laterality, or use of concomitant interventions. Rates of perioperative adverse events, including respiratory complications, were also similar between groups.

### Survival

**Fig. 2.**
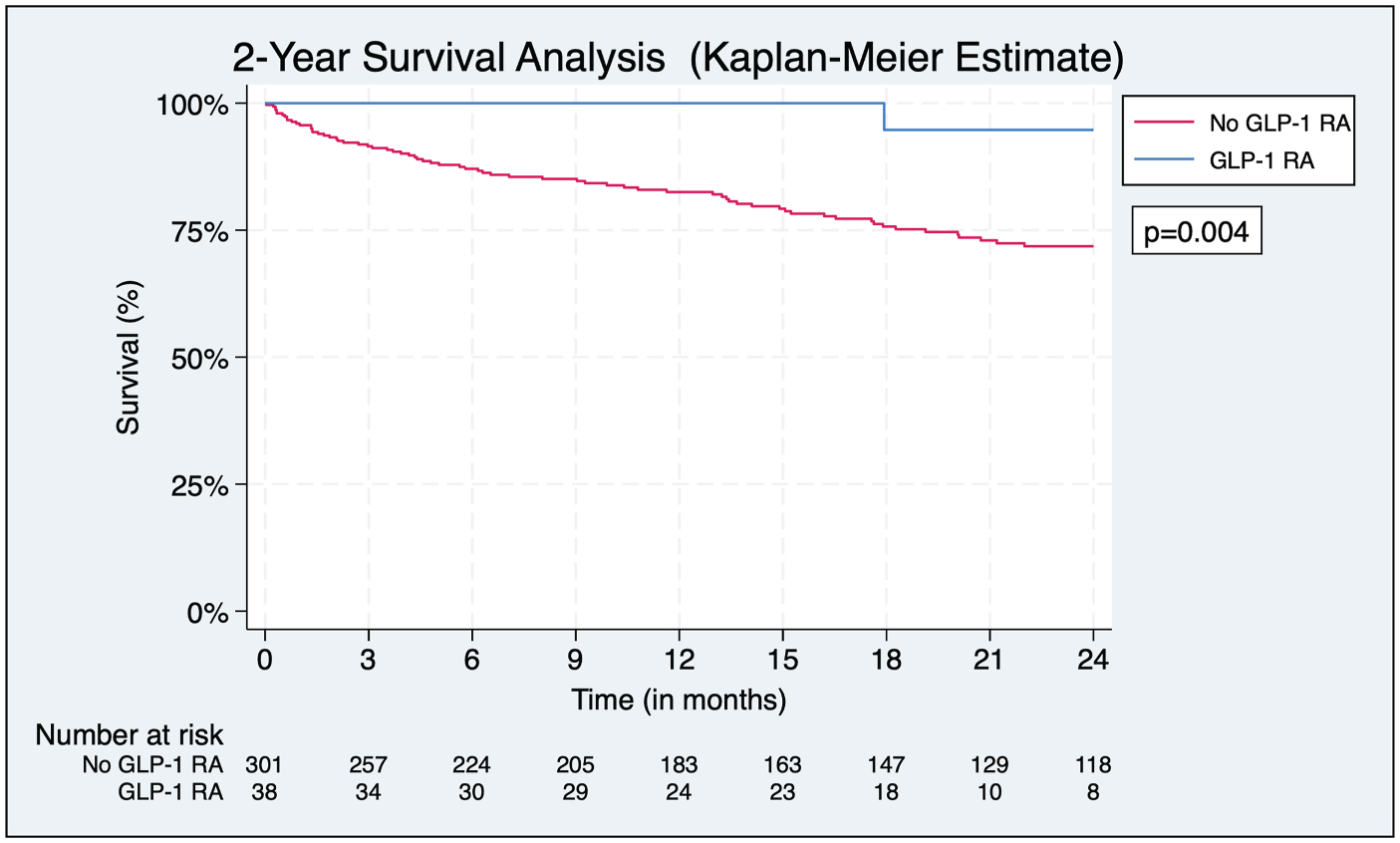
2-year postoperative survival. Kaplan–Meier survival analysis demonstrating significantly improved postoperative survival among patients on a GLP-1 RA.

**Fig. 3.**
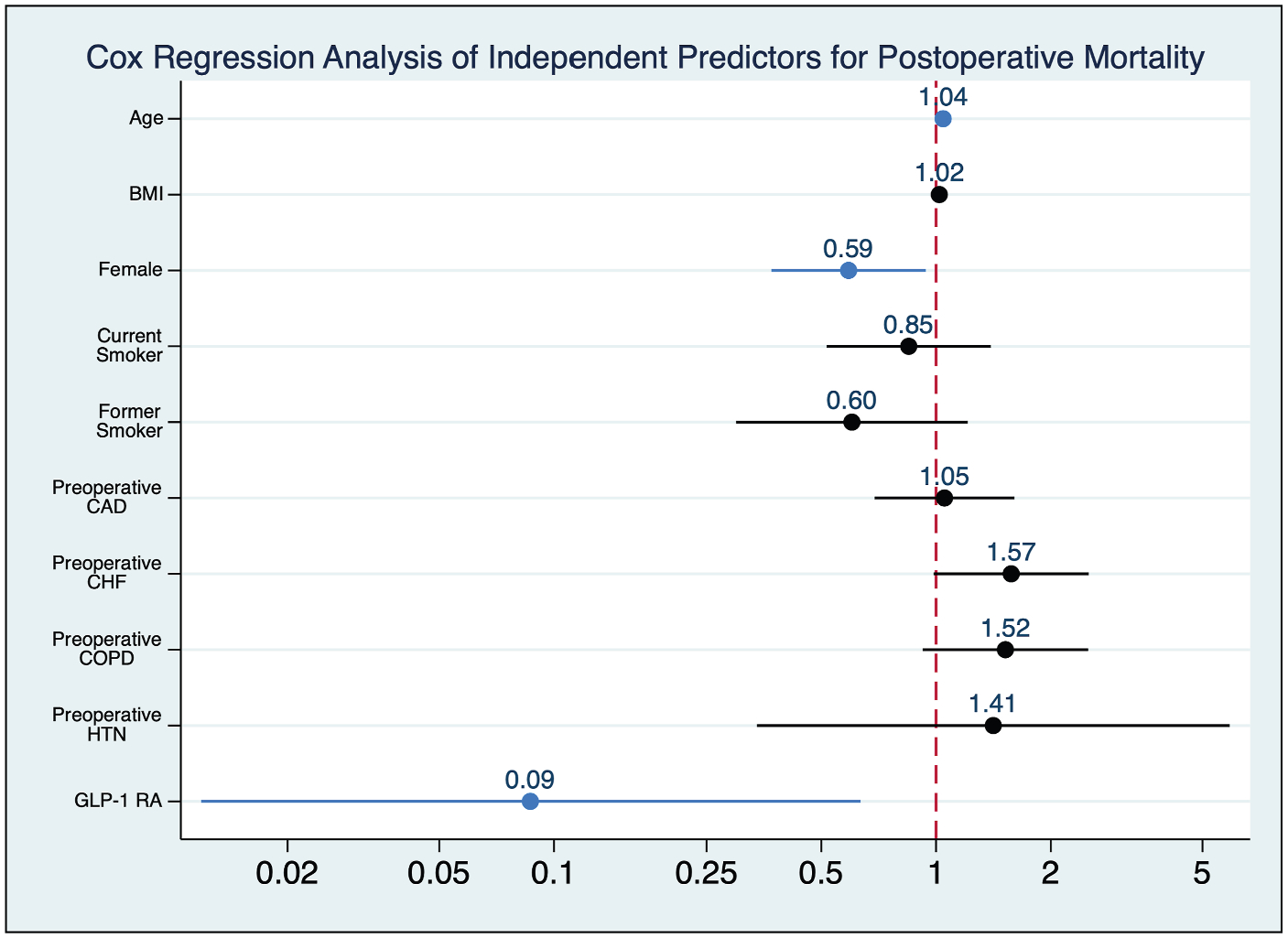
Cox regression analysis of independent predictors for postoperative mortality. GLP-1 RAs associated with a significantly reduced risk of all-cause mortality. Significance denoted by blue color. CAD, coronary artery disease; CHF, congestive heart failure; HTN, hypertension; COPD, chronic obstructive pulmonary disease.

Patients were followed for a median duration of 18^5–10,12,14–40^ months. Analysis of postoperative survival revealed that patients on a GLP-1 RA had significantly greater 2-year survival compared to those not on a GLP-1 RA (94.7% vs. 71.8%, log-rank *P* = 0.004) (Fig. 2). In the multivariate Cox regression, the use of a GLP-1 RA was independently associated with a reduced risk of all-cause mortality (aHR = 0.09, 95% CI 0.01–0.66, *P* = 0.02), even after controlling for indication (claudication vs. CLTI) (Fig. 3). Notably, comorbid conditions such as congestive heart failure and coronary artery disease were not significant predictors of survival. Lastly, 2 (0.7%) cardiovascular-related deaths occurred in the cohort, both of which were patients not on a GLP-1 RA.

The use of OMT did not differ between groups. In a subgroup analysis of patients with at least 2 components of OMT (*n* = 298, 87.9%), those on a GLP-1 RA had significantly improved 2-year survival (94.1% vs. 73.4%, *P* = 0.007). In this subgroup, multivariate Cox regression showed that GLP-1 RA use was significantly associated with a reduced risk of all-cause mortality (aHR = 0.10, 95% CI 0.01 to 0.74, *P* = 0.02).

### Major Adverse Cardiovascular Events

**Fig. 4.**
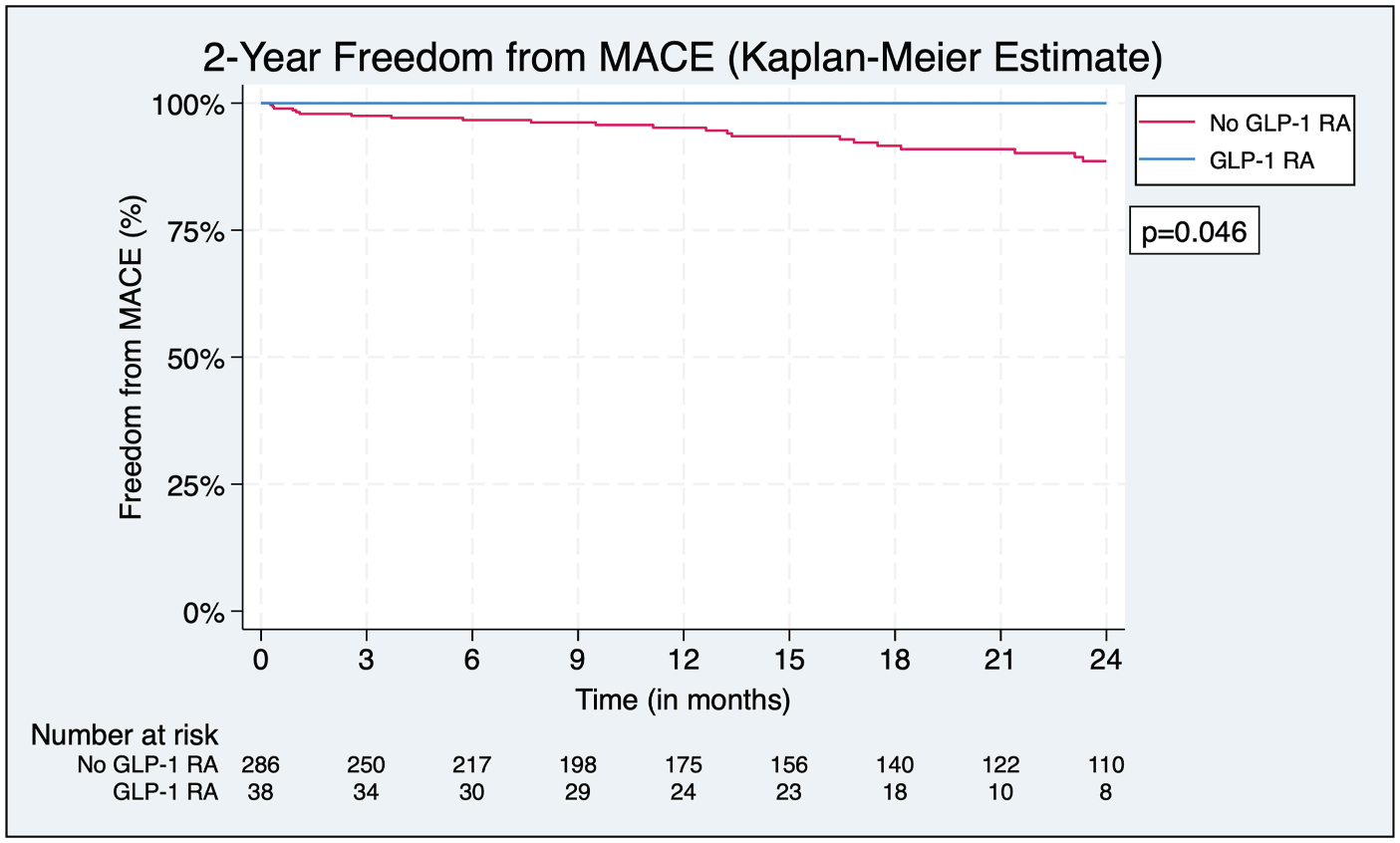
Freedom from MACE. Kaplan–Meier analysis of freedom from MACE demonstrating significantly greater freedom from MACE among patients on a GLP-1 RA.

**Fig. 5.**
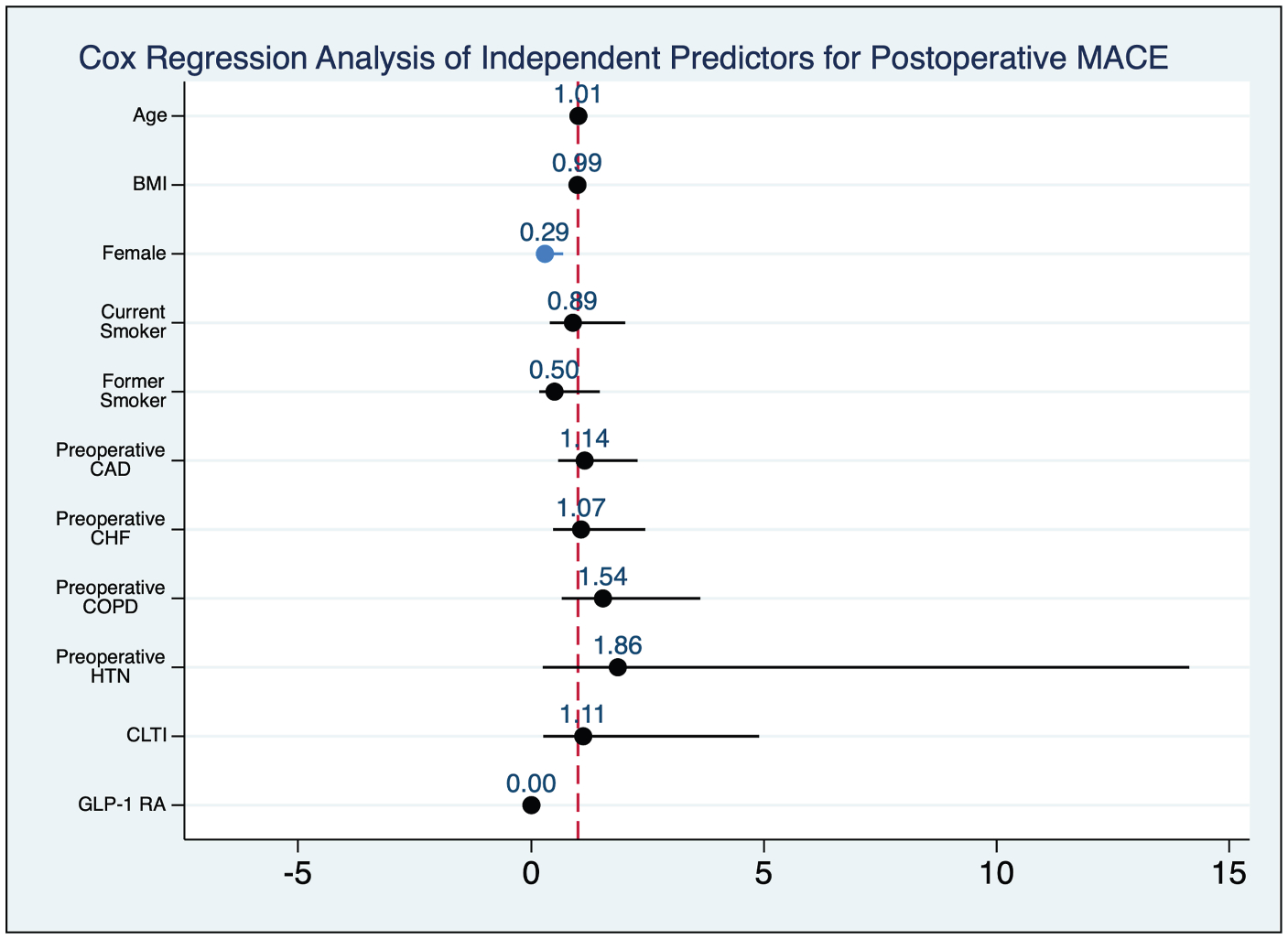
Cox Regression Analysis of Independent Predictors for Postoperative MACE. Female sex associated with a significantly reduced risk of experiencing a MACE. Significance denoted by blue color. CAD, coronary artery disease; CHF, congestive heart failure; HTN, hypertension; COPD, chronic obstructive pulmonary disease

On follow-up, patients on a GLP-1 RA had greater 2-year freedom from MACE compared to those not on a GLP-1 RA (100% vs. 88.6%, *P* = 0.046) (Fig. 4). Notably, no events were observed in the GLP-1 RA group (Table III), which precluded the calculation of an aHR for GLP-1 RA use in the multivariate Cox regression. In the model, female sex was associated with a lower risk of experiencing MACE (aHR = 0.29, 95% CI 0.12 to 0.68, *P* = 0.005) (Fig. 5).

### Major Adverse Limb Events

**Fig. 6.**
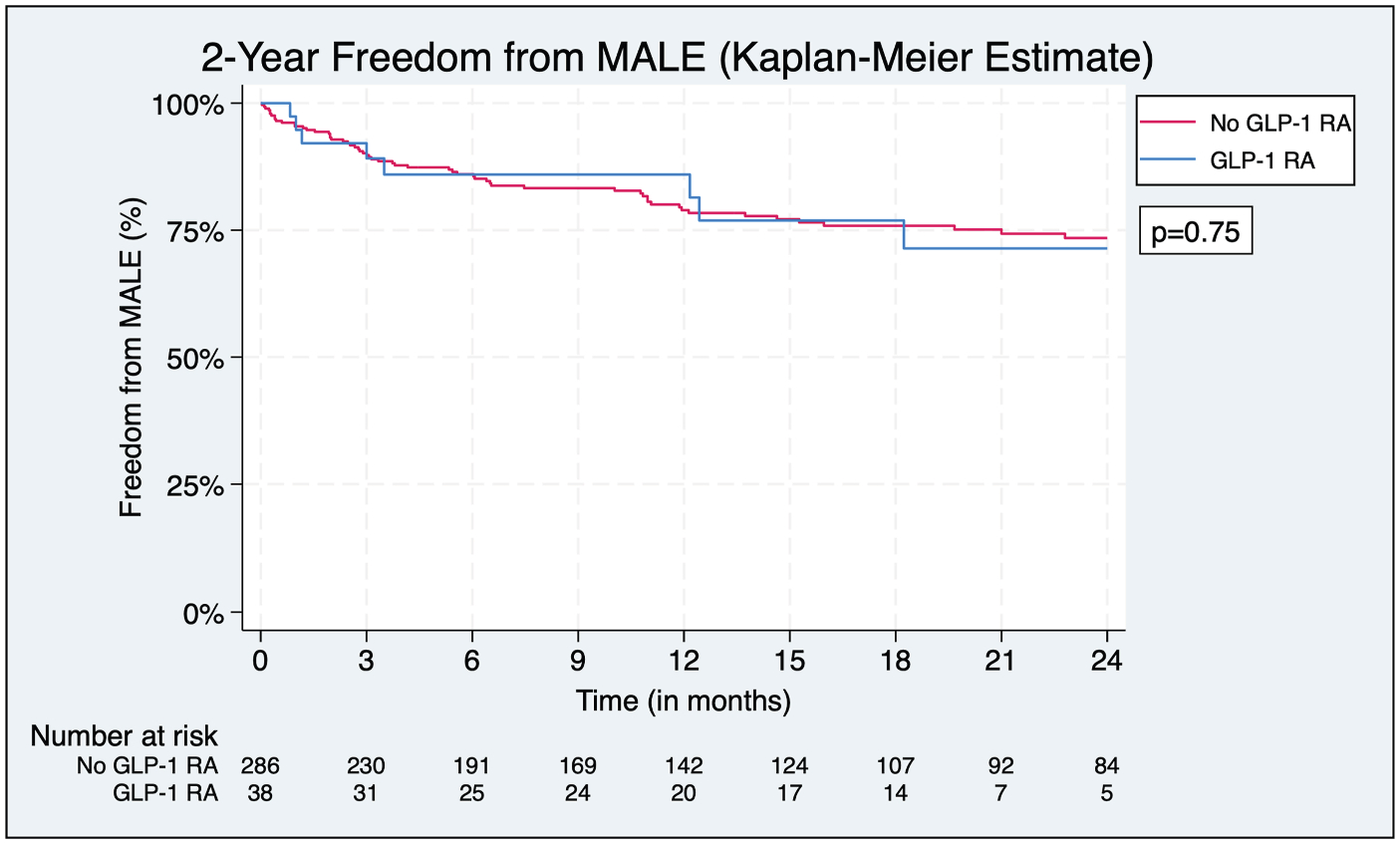
Freedom from MALE. Analysis of freedom from MALE demonstrating similar outcome between groups.

**Fig. 7.**
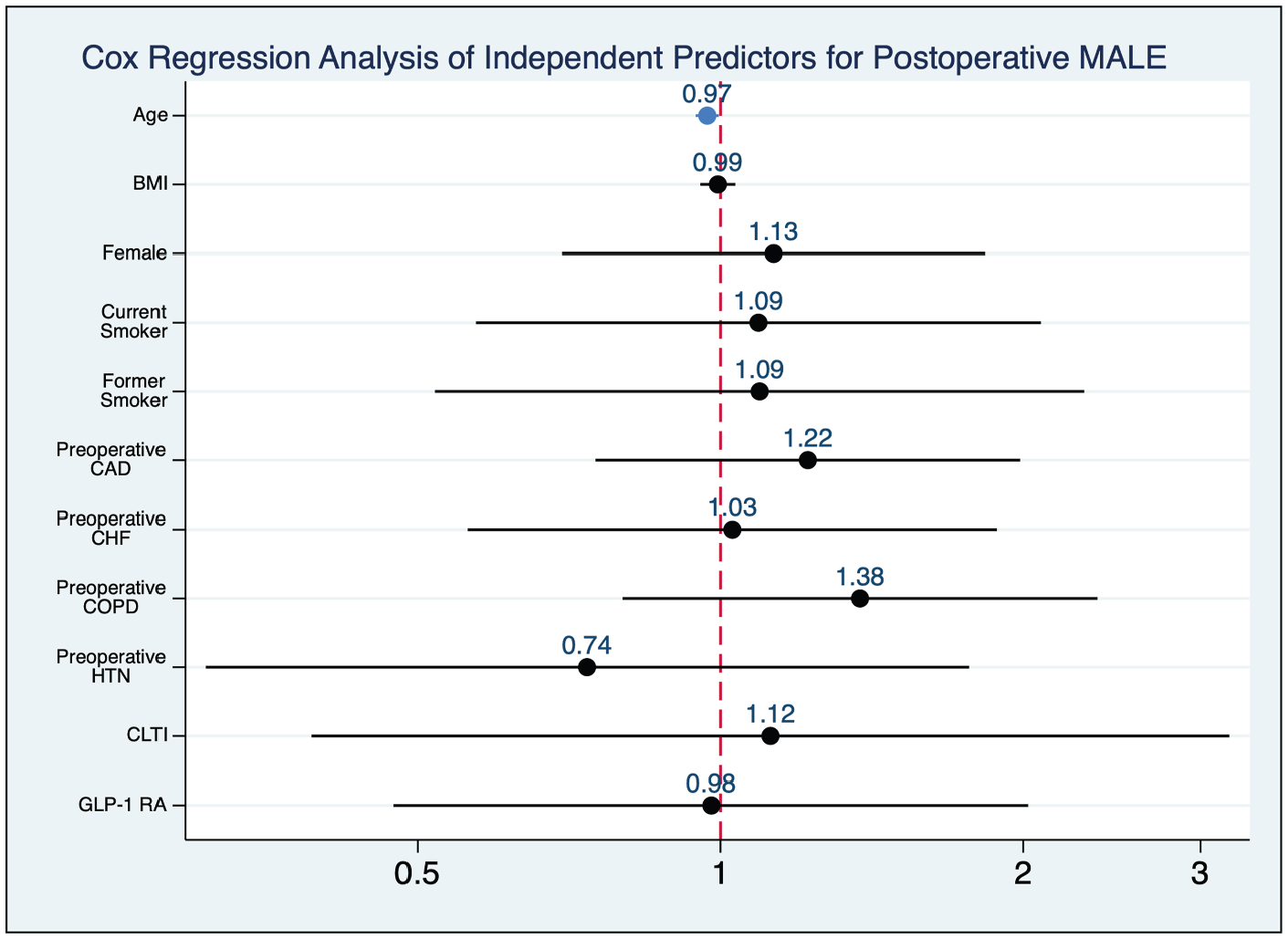
Cox regression analysis of independent predictors for postoperative MALE. Younger age associated with a significantly reduced risk of experiencing a MALE. Significance denoted by blue color. CAD, coronary artery disease; CHF, congestive heart failure; HTN, hypertension; COPD, chronic obstructive pulmonary disease.

Analysis of 2-year freedom from MALE showed no significant difference between groups (71.4% in the GLP-1 RA group vs. 73.5%, *P* = 0.75) (Fig. 6). In addition, GLP-1 RA use was not a significant predictor of MALE in the multivariate Cox regression (aHR = 0.97, 95% CI 0.47 to 1.99, *P* = 0.96). However, increasing age was associated with a decreased risk of experiencing MALE (aHR = 0.97, 95% CI 0.94 to 1.00, *P* = 0.02) (Fig. 7).

In the sensitivity analysis, MALE was redefined to include all vascular reinterventions of the index arterial segment, such as lysis, angioplasty, and/or stenting. With this broader definition, rates of MALE remained similar between groups (44.7% in the GLP-1 RA group vs. 40.9%, *P* = 0.65). Analysis of 2-year freedom from MALE also showed no significant difference (37.1% [95% CI: 16.1%–58.3%] in the GLP-1 RA group vs. 51.1% [95% CI: 44.0%–57.8%], *P* = 0.68), and GLP-1 RA use was not a significant predictor of MALE in the multivariate Cox regression (aHR = 1.05, 95% CI 0.62 to 1.79, *P* = 0.85).

### Metabolic Parameters

**Fig. 8.**
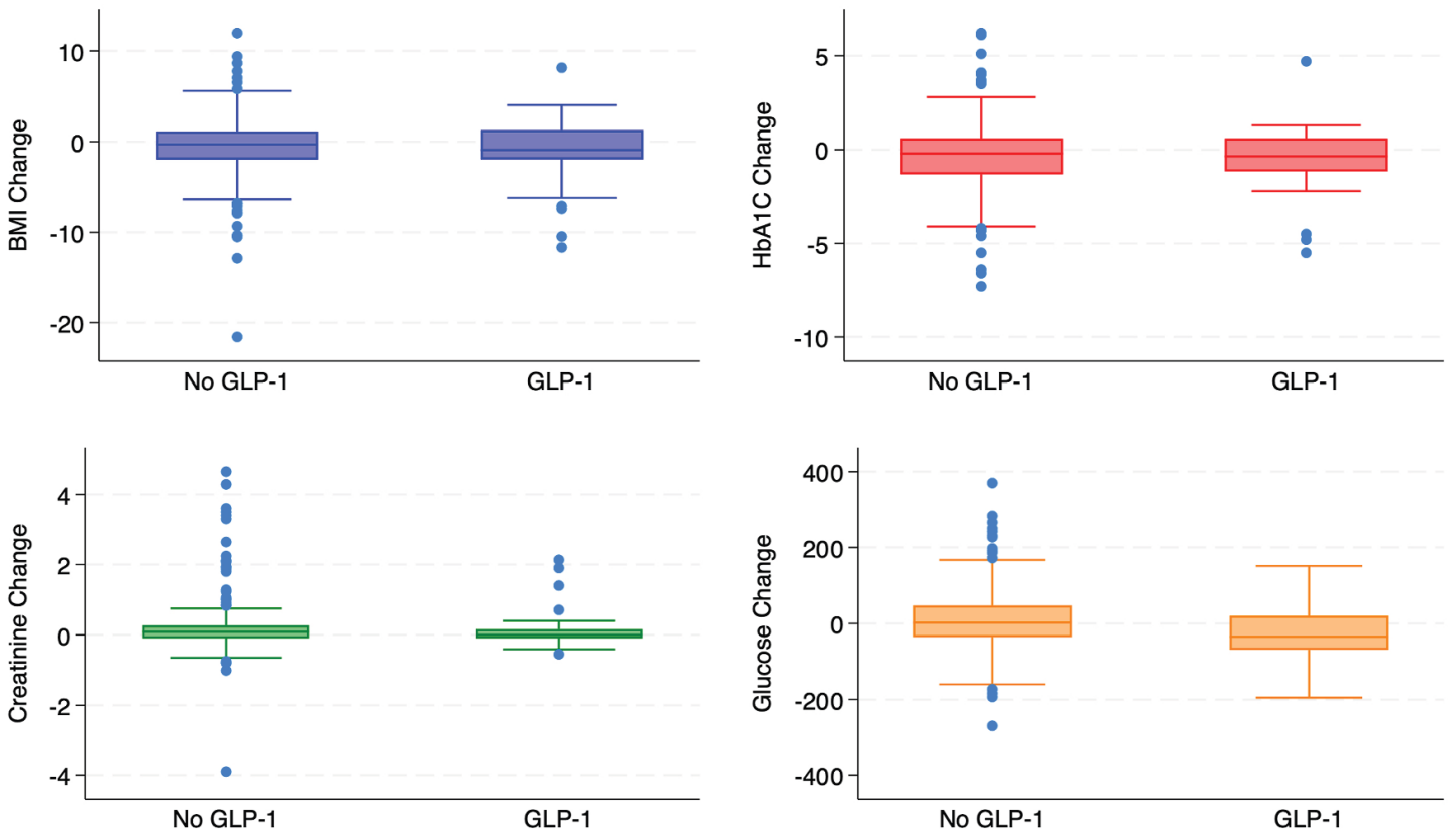
Box plot of changes in metabolic parameters. Changes in metabolic parameters by group demonstrating similar outcome between groups. HbA1C, hemoglobin A1C.

BMI, A1c, glucose, and creatinine were evaluated for changes from baseline values obtained during the preoperative assessment. Patients on a GLP-1 RA showed greater improvement across all measured parameters, although the effect was not statistically significant (Fig. 8, Table IV). Additional parameters, such as lipid levels, were excluded from the analysis due to a high degree of missing data (more than 50% missingness).

## DISCUSSION

### Improved Survival With GLP-1 RAs

In this study of adult diabetic patients undergoing infrainguinal bypass for PAD, we observed a significant association between GLP-1 RA use and improved 2-year postoperative survival. After adjusting for baseline differences, including indication for intervention, GLP-1 RAs remained independently associated with a reduced risk of all-cause mortality.^16–18^ This suggests that the survival benefit in patients on a GLP-1 RA is not attributable to differences in baseline disease severity.

Furthermore, we examined differences in OMT between groups, including the preoperative use of statins, antiplatelet agents, and nonsmoking status. OMT, a modifiable factor, is well-documented to reduce mortality and adverse cardiovascular events in patients with severe PAD.^19,20^ No significant differences in OMT were observed between the groups, and similarly, insurance status was comparable. In a subgroup analysis of patients with at least 2 of the 3 outlined components of OMT, GLP-1 RA use was associated with significantly improved survival at 2 years. This suggests that the survival benefit observed in patients on a GLP-1 RA is unlikely to be explained by the “healthy user effect” alone, meaning the benefit is not due to differences in adherence to healthier behaviors or better access to care, as the groups were comparable in these aspects.

Our finding of improved survival with GLP-1 RA use is consistent with the current literature. The Liraglutide Effect and Action in Diabetes: Evaluation of Cardiovascular Outcome Results (LEADER) trial demonstrated a 15% reduction in all-cause mortality with GLP-1 RA use compared to placebo, with a mean follow-up of 3.8 years.^5^ In this trial, the reduction in mortality was primarily driven by lower rates of adverse cardiovascular events in the GLP-1 RA group.^5^ Other CVOTs of GLP-1 RAs also showed a trend toward improved survival. A meta-analysis of 8 major CVOTs confirmed a significant survival benefit with GLP-1 RA use over a median follow-up of 3 years. In this pooled cohort of 60,080 diabetic patients, GLP-1 RA use was associated with a 12% reduction in all-cause mortality.^22^

### Reduced MACE Consistent With Findings of CVOTs

Our study found that patients on a GLP-1 RA experienced greater freedom from MACE at 2 years. Our definition of MACE was consistent with the primary outcome of 3-point MACE reported in the CVOTs, which defined MACE as a composite of cardiovascular death, nonfatal myocardial infarction, and nonfatal stroke. In our cohort, no events were observed for the composite outcome in the GLP-1 RA group. To date, 5 CVOTs have shown significant cardiovascular benefits with GLP-1 RAs. These trials, including LEADER and Semaglutide Unabated Sustainability in Treatment of Type 2 Diabetes (SUS-TAIN-6), reported notable reductions in MACE with GLP-1 RA use, including a 26% reduction in MACE with subcutaneous semaglutide in SUSTAIN-6.^8^ In the previously mentioned meta-analysis of 8 CVOTs, GLP-1 RAs reduced MACE by 14%.^22,41,42^ This effect was augmented when the Evaluation of Lixisenatide in Acute Coronary Syndrome, the only CVOT restricting enrollment to patients with recent acute coronary syndrome, was excluded.^12,22^

Overall, several important patterns emerged from these outcome trials. One key finding was that the reduction in MACE with GLP-1 RAs appeared to be influenced by the presence of established ASCVD. Across these trials, patients either had established ASCVD or ASCVD risk factors in the presence of comorbid conditions (e.g., older age, chronic kidney disease). In the LEADER trial, subgroup analysis revealed that patients with established ASCVD on a GLP-1 RA experienced greater reductions in MACE compared to those with risk factors alone.^5^ A separate study pooled data from the SUSTAIN-6 and Peptide Innovation for Early Diabetes Treatment trials to evaluate MACE-free survival with semaglutide in patients at a high ASCVD risk.^30^ This analysis found the addition of semaglutide to standard care was associated with an average increase of 1.7 life-years free of ASCVD and a reduction in 10-year ASCVD risk.^30^ Notably, patients with established ASCVD saw the greatest benefits, with larger increases in ASCVD-free life-years and greater reductions in the 10-year risk.^30^ This suggests that patients with PAD, an ASCVD equivalent, may experience larger gains in freedom from MACE compared to those with cardiovascular risk factors alone.

### Mechanism of Cardioprotection

The exact mechanism by which GLP-1 RAs exert cardioprotective effects is still unclear, but it is agreed to be multifactorial, involving metabolic, inflammatory, and atherosclerotic pathways.^31^ GLP-1 RAs are incretin mimetics that enhance postprandial insulin secretion and promote satiety through the hypothalamus, improving glycemic control.^12,32,38^ In LEADER, liraglutide significantly reduced A1c even in insulin nonresponders, and a meta-analysis found that each 1% decrease in hemoglobin A1C was associated with a 26% reduction in the MACE risk.^22^ Post hoc analyses of LEADER and SUSTAIN-6 trials found that although the reduction in MACE was consistent across participants, variations in baseline diabetes duration, BMI, and lipid levels suggested that the cardiovascular benefits of GLP-1 RAs may not be fully explained by improvements in weight and glycemic control.^32^ Instead, the authors proposed that GLP-1 RAs exert their effects mainly at the vascular level, with clinical evidence showing suppression of endothelial inflammation, inhibition of oxidative stress, and reduced plaque formation.^32^ These drugs also inhibit platelet aggregation and act as potent vasodilators.^33–35^ The delayed onset of benefits across trials further supports this mechanism, distinguishing GLP-1 RAs from sodium-glucose cotransporter 2 inhibitors, which act rapidly through diuresis and have a more pronounced effect on heart failure outcomes.^10,21,43^ In our study, patients on GLP-1 RAs showed improvements across all major metabolic parameters, although these differences were not statistically significant compared to patients not on a GLP-1 RA. However, this finding aligns with existing evidence suggesting that the cardioprotective effects of GLP-1 RAs may extend beyond glycemic control and weight reduction. Further research is necessary to understand how metabolic changes may contribute to long-term cardiovascular benefits.

### Major Adverse Limb Events

Our study did not observe a significant difference in freedom from MALE between groups at 2 years. Furthermore, GLP-1 RA use was not identified as an independent predictor of experiencing MALE on the adjusted analysis. Lastly, our sensitivity analyses showed that whether minor reinterventions were considered or not in the definition of MALE, freedom from vascular reintervention or major amputation did not differ between groups. In the current literature, the data are mixed, with several retrospective studies proposing that the use of GLP-1 RAs may improve limb outcomes. For example, a large retrospective cohort study in Taiwan, which included over a million diabetic patients treated with either a GLP-1 RA or a dipeptidyl peptidase-4 inhibitor, found that patients on a GLP-1 RA had significantly lower rates of MALE over a mean follow-up of 2 years.^44^ In patients with PAD, limb outcomes impact patient prognosis, as rates of 1-year mortality following a major amputation range from 35% to 48%.^45,46^ Although we did not demonstrate a reduction in MALE in our study, more data are needed to evaluate the role of GLP-1 RAs in potentially improving limb outcomes for patients with PAD.

### Limitations

Due to the novelty of these medications, our study has several limitations. First, follow-up duration was a limitation in the GLP-1 RA group, as a greater proportion of these patients had undergone bypass surgery in more recent years. Over the study period, only 1 death occurred in the GLP-1 RA group. This is reflected in the Kaplan–Meier analysis of survival, where the number at risk in the GLP-1 RA group is lower due to patients censored for not yet reaching their follow-up time points. To confirm the reliability of our results, we assessed the standard error at 24 months, which was <0.1 for all time-to-event outcomes, indicating that our estimates are precise and closely reflect the true survival estimates. Second, the smaller sample size in the GLP-1 RA group represents another limitation. Despite this, we were able to detect significant differences in overall survival and freedom from MACE, suggesting a treatment effect. Furthermore, our results were consistent with those from several major CVOTs and retrospective studies that have evaluated GLP-1 RAs in cardiovascular risk reduction for patients with known ASCVD and T2DM. However, for MALE, our study was likely underpowered to detect a significant difference between groups. Finally, as a retrospective chart review, our study is subject to common limitations, including reliance on the completeness of patient records and limited information on medication adherence.

## CONCLUSION

GLP1-RA use is increasingly common in patients undergoing lower extremity bypass and was associated with significantly improved postoperative survival and freedom from MACE. Further studies are required to confirm reasons for differences in outcomes.

## Figures and Tables

**Table I.** Baseline characteristics

Characteristics	No GLP-1 RA (*N* = 301)	GLP-1 RA (*N* = 38)	*P* value
Demographics			
Age, years	69.0 [62.0–77.0]	65.5 [57.0–71.0]	0.003
Male sex	189 (62.8%)	23 (60.5%)	0.86
Race			0.74
White	238 (79.1%)	29 (76.3%)	
African American	58 (19.3%)	9 (23.7%)	
Other	5 (1.6%)	0 (0.0%)	
Comorbid conditions			
CAD	151 (50.2%)	23 (60.5%)	0.30
CHF	67 (22.3%)	10 (26.3%)	0.54
HTN	286 (95.0%)	37 (97.4%)	1.00
COPD	77 (25.6%)	8 (21.1%)	0.69
Smoking			0.31
Never	73 (24.3%)	12 (31.6%)	
Prior	131 (43.5%)	18 (47.4%)	
Current	97 (32.2%)	8 (21.1%)	
Prior ipsilateral PVI	114 (37.9%)	15 (39.5%)	0.86
Metabolic parameters			
BMI, kg/m^2^	27.2 [23.3–31.1]	29.63 [26.9–33.3]	<0.001
HbA1C, mg/dL	7.4 [6.5–9.2]	6.8 [6.3–8.1]	0.14
Creatinine, mg/dL	1.06 [0.8–1.3]	0.95 [0.8–1.1]	0.19
Preoperative medications			
Antiplatelet use	121 (40.2%)	13 (34.2%)	0.60
Aspirin use	216 (71.8%)	31 (81.6%)	0.25
Statin use	252 (83.7%)	34 (89.5%)	0.48
Insurance			
Medicare	163 (54.2%)	24 (63.2%)	0.15
Medicaid	10 (3.3%)	3 (7.9%)	
Private/Commercial	119 (39.5%)	9 (23.7%)	
Other	9 (3.0%)	2 (5.2%)	
Indication			1.00
Claudication	16 (5.3%)	2 (5.3%)	
CLTI	285 (94.7%)	36 (94.7%)	

Data are presented as frequency (%), mean ± standard deviation, or median [interquartile range].

CAD, coronary artery disease; CHF, congestive heart failure; HTN, hypertension; COPD, chronic obstructive pulmonary disease; PVI, peripheral vascular intervention; HbA1C, hemoglobin A1C.

**Table II.** Operative details and in-hospital outcomes

Outcomes	No GLP-1 RA (*N* = 301)	GLP-1 RA (*N* = 38)	*P* value
Treated level			0.12
Femoral/popliteal	128 (42.5%)	11 (28.9%)	
Tibial	173 (57.5%)	27 (71.1%)	
Laterality			0.61
Right	157 (52.2%)	18 (47.4%)	
Left	144 (47.8%)	20 (52.6%)	
Concomitant Endarterectomy	77 (25.6%)	10 (26.3%)	1.00
Concomitant PVI	17 (5.6%)	4 (10.5%)	0.28
Complications			
MI	10 (3.3%)	0 (0.0%)	0.61
CHF	4 (1.3%)	0 (0.0%)	1.00
Dysrhythmia	17 (5.6%)	1 (2.6%)	0.71
Stroke	1 (0.3%)	0 (0.0%)	1.00
Respiratory	6 (2.0%)	0 (0.0%)	1.00
Renal	19 (6.3%)	2 (5.3%)	1.00
RTOR	62 (20.6%)	8 (21.1%)	1.00
Amputation	6 (2.0%)	0 (0.0%)	1.00

Data are presented as frequency (%), mean ± standard deviation, or median [interquartile range].

PVI, peripheral vascular intervention; MI, myocardial infarction, CHF, congestive heart failure; RTOR, return to operating room.

**Table III.** Outcomes on follow-up

Outcomes	No GLP-1 RA (*N* = 286)	GLP-1 RA (*N* = 38)	*P* value
Death	83 (29.0%)	1 (2.6%)	<0.001
MACE	39 (13.6%)	0 (0.0%)	0.015
MI	21 (7.3%)	0 (0.0%)	0.084
Stroke	17 (5.9%)	0 (0.0%)	0.123
Cardiovascular death	2 (0.7%)	0 (0.0%)	0.605
MALE	67 (23.4%)	9 (23.7%)	0.972
Bypass revision	46 (16.1%)	6 (15.8%)	0.963
Thrombectomy	17 (5.9%)	2 (5.3%)	0.867
Major amputation	22 (7.7%)	4 (10.5%)	0.546
Reintervention	107 (37.5%)	14 (36.8%)	0.933

Data are presented as frequency (%), mean ± standard deviation, or median [interquartile range]. Only limbs at-risk are included in *N* for each group (i.e., 286 limbs at-risk of 301 limbs in total cohort).

MI, myocardial infarction.

**Table IV.** Changes in metabolic parameters

Parameters	No GLP-1 RA (*N* = 286)	GLP-1 RA (*N* = 38)	*P* value
BMI, kg/m^2^	−0.26 [−1.96 to 1.12]	−0.91 [−1.93 to 1.26]	0.469
HbA1C, mg/dL	−0.20 [−1.30 to 0.60]	−0.35 [−1.15 to 0.60]	0.879
Creatinine, mg/dL	0.10 [−0.10 to 0.27]	−0.00 [−0.10 to 0.16]	0.188

Data are presented as median [interquartile range]. Only limbs at-risk are included in *N* for each group (i.e., 286 limbs at-risk of 301 limbs in total cohort).

HbA1C, hemoglobin A1C.

## Data Availability

Due to the size of the cohort and single-institution nature of this study, raw data cannot be publicly shared to protect patient confidentiality. However, details regarding data analysis are available on request.

## References

[1] Weng W, Kong SX, Ganguly R, The prevalence of cardiovascular disease by vascular bed and impact on healthcare costs in a large, real-world population with type 2 diabetes. Endocrinol Diabetes Metab 2020;3: e00106. 32318629 10.1002/edm2.106 PMC7170457

[2] Bevan GH, White Solaru KT. Evidence-based medical management of peripheral artery disease. Arterioscler Thromb Vasc Biol 2020;40:541–53. 31996023 10.1161/ATVBAHA.119.312142

[3] Jansen S, de Borst GJ, Hinchliffe R, Peripheral artery disease: underappreciated impact and residual cardiovascular risk despite revascularization. Clin Ther 2023;45: 1019–22. 37940497 10.1016/j.clinthera.2023.09.021

[4] Holman RR, Bethel MA, Mentz RJ, Effects of once-weekly exenatide on cardiovascular outcomes in type 2 diabetes. New Engl J Med 2017;377:1228–39. 28910237 10.1056/NEJMoa1612917 PMC9792409

[5] Marso SP, Daniels GH, Brown-Frandsen K, Liraglutide and cardiovascular outcomes in type 2 diabetes. New Engl J Med 2016;375:311–22. 27295427 10.1056/NEJMoa1603827 PMC4985288

[6] Gerstein HC, Colhoun HM, Dagenais GR, Dulaglutide and cardiovascular outcomes in type 2 diabetes (REWIND): a double-blind, randomised placebo-controlled trial. The Lancet 2019;394:121–30. 10.1016/S0140-6736(19)31149-3 31189511

[7] Husain M, Birkenfeld AL, Donsmark M, Oral semaglutide and cardiovascular outcomes in patients with type 2 diabetes. New Engl J Med 2019;381:841–51. 31185157 10.1056/NEJMoa1901118

[8] Deo SV, Marsia S, McAllister DA, The time-varying cardiovascular benefits of glucagon-like peptide-1 receptor agonist therapy in patients with type 2 diabetes mellitus: evidence from large multinational trials. Diabetes Obes Metab 2022;24:1607–16. 35491516 10.1111/dom.14738 PMC9540124

[9] Marso SP, Bain SC, Consoli A, Semaglutide and cardiovascular outcomes in patients with type 2 diabetes. New Engl J Med 2016;375:1834–44. 27633186 10.1056/NEJMoa1607141

[10] Hernandez AF, Green JB, Janmohamed S, Albiglutide and cardiovascular outcomes in patients with type 2 diabetes and cardiovascular disease (Harmony Outcomes): a double-blind, randomised placebo-controlled trial. Lancet 2018;392: 1519–29. 30291013 10.1016/S0140-6736(18)32261-X

[11] Mahé G, Aboyans V, Cosson E, Challenges and opportunities in the management of type 2 diabetes in patients with lower extremity peripheral artery disease: a tailored diagnosis and treatment review. Cardiovasc Diabetology 2024;23:220. 10.1186/s12933-024-02325-9 PMC11210102 38926722

[12] Das SR, Everett BM, Birtcher KK, 2020 expert consensus decision pathway on novel therapies for cardiovascular risk reduction in patients with type 2 diabetes: a report of the American college of Cardiology Solution Set Oversight Committee. J Am Coll Cardiol 2020;76:1117–45. 32771263 10.1016/j.jacc.2020.05.037 PMC7545583

[13] Criqui MH, Matsushita K, Aboyans V, Lower extremity peripheral artery disease: contemporary epidemiology, management gaps, and future directions: a scientific statement from the American heart association. Circulation 2021;144:e171–91. 34315230 10.1161/CIR.0000000000001005 PMC9847212

[14] Liarakos AL, Tentolouris A, Kokkinos A, Impact of Glucagon-like peptide 1 receptor agonists on peripheral arterial disease in people with diabetes mellitus: a narrative review. J Diabetes Compl 2023;37:108390. 10.1016/j.jdiacomp.2022.108390 36610322

[15] Regier EE, Venkat MV, Close KL. More than 7 years of hindsight: revisiting the fDA’s 2008 guidance on cardiovascular outcomes trials for type 2 diabetes medications. Clin Diabetes 2016;34:173–80. 27766008 10.2337/cd16-0005 PMC5070965

[16] Serra R, Bracale UM, Ielapi N, The impact of chronic kidney disease on peripheral artery disease and peripheral revascularization. Int J Gen Med 2021;14:3749–59. 34326661 10.2147/IJGM.S322417 PMC8315808

[17] Martinez OP, Storo K, Provenzano Z, A systematic review and meta-analysis on the influence of sociodemographic factors on amputation in patients with peripheral arterial disease. J Vasc Surg 2024;79:169–178.e1. 37722513 10.1016/j.jvs.2023.08.130

[18] Baubeta FE, Andersson M, Thuresson M, Editor’s choice – impact of comorbidity, medication, and gender on amputation rate following revascularisation for chronic limb threatening ischaemia. Eur J Vasc Endovasc Surg 2018;56:681–8. 30093176 10.1016/j.ejvs.2018.06.003

[19] Gornik HL, Aronow HD, Goodney PP, ACC/AHA/AACVPR/APMA/ABC/SCAI/SVM/SVN/SVS/SIR/VESS Guideline for the management of lower extremity peripheral artery disease. JACC 2024;83:2497–604. 38752899 10.1016/j.jacc.2024.02.013 PMC12728805

[20] Jarosinski MC, Hafeez MS, Sridharan ND, Markers of optimal medical therapy are associated with improved limb outcomes after elective revascularization for intermittent claudication. J Vasc Surg 2025;81:200–209.e3. 39208918 10.1016/j.jvs.2024.08.033 PMC11684783

[21] Kristensen SL, Rørth R, Jhund PS, Cardiovascular, mortality, and kidney outcomes with GLP-1 receptor agonists in patients with type 2 diabetes: a systematic review and meta-analysis of cardiovascular outcome trials. Lancet Diabetes Endocrinol 2019;7:776–85. 31422062 10.1016/S2213-8587(19)30249-9

[22] Sattar N, Lee MMY, Kristensen SL, Cardiovascular, mortality, and kidney outcomes with GLP-1 receptor agonists in patients with type 2 diabetes: a systematic review and meta-analysis of randomised trials. Lancet Diabetes Endocrinol 2021;9:653–62. 34425083 10.1016/S2213-8587(21)00203-5

[23] Sartipy F, Sigvant B, Lundin F, Ten year mortality in different peripheral arterial disease stages: a population based observational study on outcome. Eur J Vasc Endovasc Surg 2018;55:529–36. 29478910 10.1016/j.ejvs.2018.01.019

[24] Kochar A, Mulder H, Rockhold FW, Cause of death among patients with peripheral artery disease. Circ Cardiovasc Qual Outcomes 2020;13:e006550. 33176462 10.1161/CIRCOUTCOMES.120.006550

[25] Soyoye DO, Abiodun OO, Ikem RT, Diabetes and peripheral artery disease: a review. World J Diabetes 2021;12:827–38. 34168731 10.4239/wjd.v12.i6.827 PMC8192257

[26] Verma S, Leiter LA, Mangla KK, Epidemiology and burden of peripheral artery disease in people with type 2 diabetes: a systematic literature review. Diabetes Ther 2024;15:1893–961. 39023686 10.1007/s13300-024-01606-6 PMC11330435

[27] Morrison JT, Canonico ME, Anand SS, Low-dose rivaroxaban plus aspirin in patients with peripheral artery disease undergoing lower extremity revascularization with and without concomitant coronary artery disease: insights from VOYAGER PAD. Circulation 2024;149:1536–9. 38709838 10.1161/CIRCULATIONAHA.124.068080

[28] Debus ES, Nehler MR, Govsyeyev N, Effect of rivaroxaban and aspirin in patients with peripheral artery disease undergoing surgical revascularization: insights from the VOYAGER PAD trial. Circulation 2021;144:1104–16. 34380322 10.1161/CIRCULATIONAHA.121.054835

[29] Gerstein HC, Sattar N, Rosenstock J, Cardiovascular and renal outcomes with efpeglenatide in type 2 diabetes. New Engl J Med 2021;385:896–907. 34215025 10.1056/NEJMoa2108269

[30] Westerink J, Matthiessen KS, Nuhoho S, Estimated life-years gained free of new or recurrent major cardiovascular events with the addition of semaglutide to standard of care in people with type 2 diabetes and high cardiovascular risk. Diabetes Care 2022;45:1211–8. 35263432 10.2337/dc21-1138 PMC9174968

[31] Lincoff AM, Brown-Frandsen K, Colhoun HM, Semaglutide and cardiovascular outcomes in obesity without diabetes. New Engl J Med 2023;389:2221–32. 37952131 10.1056/NEJMoa2307563

[32] Park B, Bakbak E, Teoh H, GLP-1 receptor agonists and atherosclerosis protection: the vascular endothelium takes center stage. Am J Physiol 2024;326:H1159–76. 10.1152/ajpheart.00574.2023 38426865

[33] Song X, Jia H, Jiang Y, Anti-atherosclerotic effects of the glucagon-like peptide-1 (GLP-1) based therapies in patients with type 2 diabetes mellitus: a meta-analysis. Scientific Rep 2015;5:10202. 10.1038/srep10202 PMC4481643 26111974

[34] Jia G, Aroor AR, Sowers JR. Glucagon-like peptide 1 receptor activation and platelet function: beyond glycemic control. Diabetes 2016;65:1487–9. 27222394 10.2337/dbi16-0014 PMC4878428

[35] Xie D, Li Y, Xu M, Effects of dulaglutide on endothelial progenitor cells and arterial elasticity in patients with type 2 diabetes mellitus. Cardiovasc Diabetol 2022;21: 200. 36199064 10.1186/s12933-022-01634-1 PMC9533545

[36] Koska J, Sands M, Burciu C, Exenatide protects against Glucose- and lipid-induced endothelial dysfunction: evidence for direct vasodilation effect of GLP-1 receptor agonists in humans. Diabetes 2015;64:2624–35. 25720388 10.2337/db14-0976 PMC4477348

[37] Zhang J, Xian T-Z, Wu M-X, Comparison of the effects of twice-daily exenatide and insulin on carotid intima-media thickness in type 2 diabetes mellitus patients: a 52-week randomized, open-label, controlled trial. Cardiovasc Diabetol 2020;19:48. 32334592 10.1186/s12933-020-01014-7 PMC7183674

[38] Zheng Z, Zong Y, Ma Y, Glucagon-like peptide-1 receptor: mechanisms and advances in therapy. Signal Transd Target Ther 2024;9:234. 10.1038/s41392-024-01931-z PMC11408715 39289339

[39] Cameron-Vendrig A, Reheman A, Siraj MA, Glucagon-like peptide 1 receptor activation attenuates platelet aggregation and thrombosis. Diabetes 2016;65:1714–23. 26936963 10.2337/db15-1141

[40] Longato E, Di Camillo B, Sparacino G, Cardiovascular effectiveness of human-based vs. exendin-based glucagon like peptide-1 receptor agonists: a retrospective study in patients with type 2 diabetes. Eur J Prev Cardiol 2020;28: 22–9. 10.1093/eurjpc/zwaa081 33624059

[41] Bonaca MP, Catarig AM, Hansen Y, Design and baseline characteristics of the STRIDE trial: evaluating semaglutide in people with symptomatic peripheral artery disease and type 2 diabetes. Eur Heart J Cardiovasc Pharmacother 2025;10:728–37. 39424598 10.1093/ehjcvp/pvae071 PMC11724141

[42] Felix N, Gauza MM, Bittar V, Cardiovascular and kidney outcomes of glucagon-like peptide 1 receptor agonist therapy in type 2 diabetes mellitus and chronic kidney disease: a systematic review and meta-analysis. Cardiorenal Med 2025;15:98–107. 39746343 10.1159/000543149 PMC11844710

[43] Baviera M, Genovese S, Lepore V, Lower risk of death and cardiovascular events in patients with diabetes initiating glucagon-like peptide-1 receptor agonists or sodium-glucose cotransporter-2 inhibitors: a real-world study in two Italian cohorts. Diabetes Obes Metab 2021;23:1484–95. 33606897 10.1111/dom.14361

[44] Lin DS-H, Lee J-K, Chen W-J. Major adverse cardiovascular and limb events in patients with diabetes treated with GLP-1 receptor agonists vs DPP-4 inhibitors. Diabetologia 2021;64: 1949–62. 34195865 10.1007/s00125-021-05497-1

[45] Abry L, Weiss S, Makaloski V, Peripheral artery disease leading to major amputation: trends in revascularization and mortality over 18 years. Ann Vasc Surg 2022;78: 295–301. 34182110 10.1016/j.avsg.2021.04.037

[46] Long CA, Mulder H, Fowkes FGR, Incidence and factors associated with major amputation in patients with peripheral artery disease. Circ Cardiovasc Qual Outcomes 2020;13:e006399. 32615798 10.1161/CIRCOUTCOMES.119.006399

